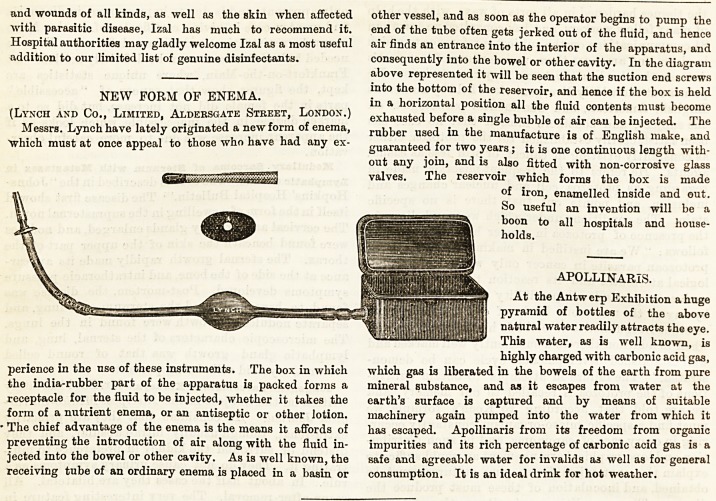# New Appliances and Things Medical

**Published:** 1894-06-30

**Authors:** 


					NEW APPLIANCES AND THINGS JYIEDICAL.
[All preparations, appliances, novelties, &c., of which a notice is desired, should be sent for the Editor, to care of The Manager, 428,
Strand, London, W.0.1
IZAL?A NEW ANTISEPTIC
(Newton, Chambers and Co., Thorncliffe.)
.This new antiseptic which in a comparatively short time
has made considerable headway in public favour, is a non
poisonous creamy liquid. Although the active antiseptic
principle is not itself soluble in water the disinfectant can be
mixed with water in all proportions forming a milky solution
with the active principle in suspension and intimate admixture
with the fluid medium. Its chief uses are for washing down
the furniture, walls, floors. &c. of infected rooms. For this
purpose one tablespoon of Izal with a pint of water is a
sufficiently powerful solution. In the same manner contami-
nated linen or clothing may be disinfected. After scarlatina
or other of the exanthemata the patient may have a bath in
which two tablespoonfuls of Izal to the 20 gallons of water
has been added ; and for direct application to cuts, bruises,.
280 THE HOSPITAL.
June 30, 1894.
and wounds of all kinds, as well as the skin when affected
with parasitic disease, Izal has much to recommend it.
Hospital authorities may gladly welcome Izal as a most useful
addition to our limited list of genuine disinfectants.
NEW FORM OF ENEMA.
(Lynch and Co., Limited, Aldersgate Street, London.)
Messrs. Lynch have lately originated a new form of enema,
"which must at once appeal to those who have had any ex-
perience in the use of these instruments. The box in which
the india-rubber part of the apparatus is packed forms a
receptacle for the fluid to be injected, whether it takes the
form of a nutrient enema, or an antiseptic or other lotion.
The chief advantage of the enema is the means it affords of
preventing the introduction of air along with the fluid in-
jected into the bowel or other cavity. As is well known, the
receiving tube of an ordinary enema is placed in a basin or
other vessel, and as soon as the operator begins to pump the
end of the tube often gets jerked out of the fluid, and hence
air finds an entrance into the interior of the apparatus, and
consequently into the bowel or other cavity. In the diagram
above represented it will be seen that the suction end screws
into the bottom of the reservoir, and hence if the box is held
in a horizontal position all the fluid contents must become
exhausted before a single bubble of air can be injected. The
rubber used in the manufacture is of English make, and
guaranteed for two years; it is one continuous length with-
out any join, and is also fitted with non-corrosive glass
valves. The reservoir which forms the box is made
of iron, enamelled inside and out.
So useful an invention will be a
boon to all hospitals and house-
holds.
APOLLINARIS.
At the Antw erp Exhibition a huge
pyramid of bottles of the above
natural water readily attracts the eye.
This water, as is well known, is
highly charged with carbonic acid gas,
which gas is liberated in the bowels of the earth from pure
mineral substance, and as it escapes from water at the
earth's surface is captured and by means of suitable
machinery again pumped into the water from which it
has escaped. Apollinaris from its freedom from organic
impurities and its rich percentage of carbonic acid gas is a
safe and agreeable water for invalids as well as for general
consumption. It is an ideal drink for hot weather.
and wounds of all kinds, as well as the skin when affected
with parasitic disease, Izal has much to recommend it.
Hospital authorities may gladly welcome Izal as a most useful
addition to our limited list of genuine disinfectants.
NEW FORM OF ENEMA.
(Lynch and Co., Limited, Aldersgate Street, London.)
Messrs. Lynch have lately originated a new form of enema,
"which must at once appeal to those who have had any ex-
perience in the use of these instruments. The box in which
the india-rubber part of the apparatus is packed forms a
receptacle for the fluid to be injected, whether it takes the
form of a nutrient enema, or an antiseptic or other lotion.
The chief advantage of the enema is the means it affords of
preventing the introduction of air along with the fluid in-
jected into the bowel or other cavity. As is well known, the
receiving tube of an ordinary enema is placed in a basin or
other vessel, and as soon as the operator begins to pump the
end of the tube often gets jerked out of the fluid, and hence
air finds an entrance into the interior of the apparatus, and
consequently into the bowel or other cavity. In the diagram
above represented it will be seen that the suction end screws
into the bottom of the reservoir, and hence if the box is held
in a horizontal position all the fluid contents must become
exhausted before a single bubble of air cau be injected. The
rubber used in the manufacture is of English make, and
guaranteed for two years; it is one continuous length with-
out any join, and is also fitted with non-corrosive glass
valves. The reservoir which forms the box is made
of iron, enamelled inside and out.
So useful an invention will be a
boon to all hospitals and house-
holds.
APOLLINARIS.
At the Antwerp Exhibition a huge
pyramid of bottles of the above
natural water readily attracts the eye.
This water, as is well known, is
highly charged with carbonic acid gas,
which gas is liberated in the bowels of the earth from pure
mineral substance, and as it escapes from water at the
earth's surface is captured and by means of suitable
machinery again pumped into the water from which it
has escaped. Apollinaris from its freedom from organic
impurities and its rich percentage of carbonic acid gas is a
safe and agreeable water for invalids as well as for general
consumption. It is an ideal drink for hot weather.

				

## Figures and Tables

**Figure f1:**